# Developmental milestones in early childhood and genetic liability to neurodevelopmental disorders

**DOI:** 10.1017/S0033291721003330

**Published:** 2023-04

**Authors:** Laurie J. Hannigan, Ragna Bugge Askeland, Helga Ask, Martin Tesli, Elizabeth Corfield, Ziada Ayorech, Per Magnus, Pål Rasmus Njølstad, Anne-Siri Øyen, Camilla Stoltenberg, Ole A. Andreassen, Angelica Ronald, George Davey Smith, Ted Reichborn-Kjennerud, Alexandra Havdahl

**Affiliations:** 1 Nic Waals Institute, Lovisenberg Diaconal Hospital, Oslo, Norway; 2 MRC Integrative Epidemiology Unit, Bristol Medical School, University of Bristol, Bristol, UK; 3 Department of Mental Disorders, Norwegian Institute of Public Health, Oslo, Norway; 4 NORMENT Centre, Institute of Clinical Medicine, University of Oslo, Oslo, Norway; 5 Centre for Fertility and Health, Norwegian Institute of Public Health, Oslo, Norway; 6 Institute of Health and Society, University of Oslo, Oslo, Norway; 7 Department of Clinical Science, KG Jebsen Center for Diabetes Research, University of Bergen, Bergen, Norway; 8 Department of Pediatrics and Adolescents, Haukeland University Hospital, Bergen, Norway; 9 Norwegian Institute of Public Health, Oslo, Norway; 10 Department of Global Public Health and Primary Care, University of Bergen, Bergen, Norway; 11 Division of Mental Health and Addiction, Oslo University Hospital, Oslo, Norway; 12 Department of Psychological Sciences, Centre for Brain and Cognitive Development, Birkbeck, University of London; 13 Institute of Clinical Medicine, University of Oslo, Oslo, Norway; 14 Department of Psychology, Promenta Research Center, University of Oslo, Oslo, Norway

**Keywords:** Autism, ADHD, schizophrenia, neurodevelopment, polygenic score, motor, language

## Abstract

**Background:**

Timing of developmental milestones, such as age at first walking, is associated with later diagnoses of neurodevelopmental disorders. However, its relationship to *genetic risk* for neurodevelopmental disorders in the general population is unknown. Here, we investigate associations between attainment of early-life language and motor development milestones and genetic liability to autism, attention deficit hyperactivity disorder (ADHD), and schizophrenia.

**Methods:**

We use data from a genotyped sub-set (*N* = 25699) of children in the Norwegian Mother, Father and Child Cohort Study (MoBa). We calculate polygenic scores (PGS) for autism, ADHD, and schizophrenia and predict maternal reports of children's age at first walking, first words, and first sentences, motor delays (18 months), and language delays and a generalised measure of concerns about development (3 years). We use linear and probit regression models in a multi-group framework to test for sex differences.

**Results:**

We found that ADHD PGS were associated with earlier walking age (*β* = −0.033, *p_adj_* < 0.001) in both males and females. Additionally, autism PGS were associated with later walking (*β* = 0.039, *p*_adj_ = 0.006) in females only. No robust associations were observed for schizophrenia PGS or between any neurodevelopmental PGS and measures of language developmental milestone attainment.

**Conclusions:**

Genetic liabilities for neurodevelopmental disorders show some specific associations with the age at which children first walk unsupported. Associations are small but robust and, in the case of autism PGS, differentiated by sex. These findings suggest that early-life motor developmental milestone attainment is associated with genetic liability to ADHD and autism in the general population.

## Introduction

Children's timely attainment of developmental milestones is an important indicator of the extent to which early neurodevelopment is progressing typically (Bishop, Thurm, Farmer, & Lord, 2016; Johnson, Gliga, Jones, & Charman, 2015; Mayes & Calhoun, 2003). It is well established that both language and motor delays in early-life predict subsequent diagnoses of autism spectrum disorder (Johnson et al., 2015; Jones, Gliga, Bedford, Charman, & Johnson, 2014; hereafter we use preferred term ‘autism’, as per Kenny et al., 2015). Evidence is less consistent regarding associations between early developmental milestones and attention deficit hyperactivity disorder (ADHD; Havmoeller, Thomsen, & Lemcke, 2018; Mitchell *et al*. 2006), whereas delays in attaining early motor milestones do appear to be associated with diagnoses of schizophrenia later in life (Filatova et al., 2017; Isohanni et al., 2001). Given that each of these disorders has been shown to be substantially genetic in origin (ADHD: Demontis *et al*. 2019; Khan & Faraone, 2006; autism: Grove *et al*. 2019; Waye & Cheng, 2018; schizophrenia: Birnbaum & Weinberger, 2017; PGC Schizophrenia Working Group, 2014), it is plausible that links to early-life developmental milestones are underpinned by shared genes.

Autism is typically diagnosed during early childhood and is often (though not always) associated with language and motor difficulties. There is substantial evidence that delays in motor development (Harris, 2017; West, 2019) and language (Johnson et al., 2015; Jones et al., 2014) predicts subsequent autism diagnoses. Autism shares some features with ADHD, another childhood-onset neurodevelopmental condition, but ADHD is typically diagnosed in mid-childhood or later (Kessler et al., 2007). Delays in language development have been shown to predict ADHD diagnoses, but these may partly be accounted for by co-occurring autism (Johnson et al., 2015). For early motor development, the directions of documented associations with ADHD are inconsistent (Havmoeller et al., 2018). Schizophrenia, although typically not diagnosed until early adulthood (Jones, 2013), is also increasingly conceptualised as a neurodevelopmental disorder (Lewis & Levitt, 2002). This conceptualisation is supported both by evidence of some clinical overlap with disorders such as autism and ADHD (De Crescenzo et al., 2019), and also by associations between delays in the attainment of early developmental milestones and later schizophrenia diagnoses (Filatova et al., 2017; Isohanni et al., 2001; Sørensen et al., 2010; Stochl et al., 2019).

Individual differences in liability to neurodevelopmental disorders are partly genetic in origin (Lichtenstein, Carlström, Råstam, Gillberg, & Anckarsäter, 2010). Although some of the genetic variants involved occur at low frequencies in the population, much of the genetic risk for neurodevelopmental disorders is conferred by a vast number of common single-nucleotide polymorphisms (SNPs) distributed across the genome. These SNPs have small effects individually, but cumulatively explain a substantial proportion of variance in the risk for neurodevelopmental disorders [SNP heritability of 45% in schizophrenia, 22% in ADHD and 12% in autism; (Sullivan et al., 2018)]. Recently, genome-wide association studies (GWAS) have begun to identify specific SNPs conferring increased risk for neurodevelopmental disorders. In the case of autism, the most recent GWAS (Grove et al., 2019) of ~18 000 cases and ~ 28 000 controls found five independent genome-wide significant loci, while for ADHD (Demontis et al., 2019; ~ 20 000 cases; ~ 35 000 controls) 12 independent loci were found. Sample sizes for both of these traits remain relatively small in GWAS terms, whereas schizophrenia is one of the most comprehensively studied psychiatric disorders in a GWAS context, with a sample size of more than 69,000 cases and 236,000 controls yielding 270 genome wide significant loci (The Schizophrenia Working Group of the Psychiatric Genomics Consortium, Ripke, Walters, & O’Donovan, 2020). By aggregating the effects of these loci, along with weaker effects of many other SNPs across the genome, into an individual-level *polygenic score*, it is possible to explore the manifestations of genetic risk for neurodevelopmental disorders in non-clinical populations (Wray et al., 2014).

It is plausible that shared genetic factors underpin observed links between neurodevelopmental disorders and early motor and language development, both of which are also heritable (Dale et al., 1998; Smith et al., 2017). This principle is demonstrated by evidence that rare protein-truncating genetic variants implicated in autism risk are associated with delayed walking and global developmental delays (Satterstrom et al., 2020). Polygenic approaches have only recently begun to be applied in this area (Thapar, 2020). Across two studies, Serdarevic and colleagues showed that genetic liability to schizophrenia (Serdarevic et al., 2018) and autism (but not ADHD; Serdarevic et al., 2020) are associated with less advanced overall infant motor development measured between 2 and 5 months of age. A recent study in a sample of Japanese children reported a similar association between autism polygenic scores (PGS) and gross motor delay at 18 months, as well as a link with receptive (but not expressive) language delay (Takahashi et al., 2020). Despite the popularity of the ‘neurodevelopmental hypothesis’ of schizophrenia, and widespread application of schizophrenia PGS in analyses of early childhood behavioural measures (e.g. Jansen *et al*. 2018; Riglin *et al*. 2017), we are aware of no published analyses of schizophrenia PGS and early motor and language developmental milestones.

In the current study, we consider both typical and delayed motor and language development in the context of common genetic risk for autism, ADHD, and schizophrenia. We have two related aims. First, we aim to establish whether there are linear relationships between the extent of individuals' genetic ‘load’ of neurodevelopmental disorder-associated alleles and the age at which they first walk and talk. Second, we aim to establish whether there are probabilistic relationships between the same indices of genetic risk and the likelihood of motor or language delays during early childhood. With neurodevelopmental disorders diagnosed in males at a substantially higher rate than in females, we also test for sex differences in these relationships.

## Methods

### Participants and procedure

The Norwegian Mother, Father and Child Cohort Study (MoBa; Magnus *et al*. 2006, 2016) is a population-based pregnancy cohort study conducted by the Norwegian Institute of Public Health. Participants were recruited from all over Norway from 1999 to 2008. The women consented to participation in 41% of the pregnancies. The cohort now includes 114 500 children, 95 200 mothers and 75 200 fathers. The current study is based on version 12 of the quality-assured data files released for research in January 2019.

The establishment of and data collection in MoBa was previously based on a license from the Norwegian Data Protection Agency and approval from The Regional Committees for Medical and Health Research Ethics and it is now based on regulations related to the Norwegian Health Registry Act. The current study was approved by The Regional Committees for Medical and Health Research Ethics (2016/1702).

#### Genotype data

In MoBa, blood samples were obtained from children (umbilical cord) at birth (Rønningen et al., 2006). At the time of writing, genotyping of the entire MoBa cohort is ongoing, and the available sample at the time of analysis was used. Quality control procedures used to process the genotype data are outlined in online Supplementary eMethods 1. After quality control and exclusions on the basis of sample overlap,^†^^1^ a core sample of ethnically homogeneous, unrelated children (*N* = 25·699) with a high genotype call-rate (>95%) and 7 141 482 SNPs at a minor allele frequency >1% were identified for use in our analysis.

### Measures

#### Polygenic scores

We calculated PGS using PRSice2 (Choi & O'Reilly, 2019), as weighted sums of risk alleles per individual, with weights based on effect sizes in European samples in the most recent GWAS of, respectively, ADHD (Demontis et al., 2019), autism spectrum disorders (Grove et al., 2019; referred to here as autism), and schizophrenia (The Schizophrenia Working Group of the Psychiatric Genomics Consortium, Ripke, Walters, & O'Donovan, 2020). Separate PGS were calculated using 10 nested sets of SNPs, with a SNP included in the first score only if the *p* value of its association in the original GWAS was < 5 × 10^−8^ (the commonly applied ‘genome-wide significance threshold’), then in the next score if *p* < 1 × 10^−6^, and so on through a further eight thresholds (1 ×  10^−5^. 1 × 10^−4^. 0.001, 0.005, 0.01, 0.1, 0.5) culminating in one (*p* < 1) with all SNPs included. Although the specific thresholds are selected somewhat arbitrarily, the approach in general aims to maximise the polygenic signal by including increasing numbers of SNPs with increasingly weak associations, with the assumption that prediction will continue to increase until additional SNPs contribute only statistical noise to the score. All PGS were regressed on genotype batch and 10 principal components to account for possible structural artefacts in the data. Next, in order to have a single polygenic predictor per neurodevelopmental trait in the analyses, we extracted the first principal component from analyses of scores at all 10 thresholds for a given phenotype – an approach recently shown by Coombes, Ploner, Bergen, and Biernacka (2020) to maximise prediction while reducing the risk of over-fitting. Full details of the parameters used in generating the PGS and correlations between the PGS at each threshold and the principal component score (henceforth: the PGS) used in these analyses are presented in online Supplementary eMethods 2.

#### Motor development

Mothers reported on the age (in months) at which their child first walked unsupported at two waves of data collection: when children were 18 months old and 3 years old. We constructed a single age-at-first-walking variable for analysis by taking available data from either the 18-month or 3-year-old version if only one was available, and by calculating a mean (rounded to the nearest month) if both were available [these measures are highly correlated in the full MoBa sample (*r* = 0.84, *n* = 50 940)]. Values considered to be implausible (i.e. children reported as walking at <6 months) were excluded.

In addition to the continuous age-at-first-walking measure, we derived a binary measure relating to the presence or absence of motor delays. This was based on three items included in the questionnaire completed by mothers when their children were 18 months old. The first simply asked whether or not children were yet able to walk unaided, and two items from the Ages and Stages Questionnaire (ASQ; Squires, LaWanda, and Bricker, 1999); for validation of the Norwegian version see: Richter and Janson, 2007) asked mothers to report whether their child ‘can walk well and seldom fall’, ‘moves around by walking, rather than by crawling on his/her hands and knees’. If mothers responded No/Not yet to any of these items, we coded the child as having signs of delayed motor abilities at 18 months. Details of internal validation checks of this derived measure are presented in online Supplementary eMethods 3.

#### Language development

When the children were 5 years old, mothers reported on the age (in months) at which their child used their first single words and, separately, their first sentences (putting together 2–3 words). Values considered to be implausible (i.e. children reported as talking at <6 months) were excluded. By subtracting children's age at first words from their age at first sentence use, we also calculated a variable indexing the rate of language learning.

In addition to the continuous language development measures, we also constructed a binary measure relating to the presence or absence of delays in language abilities. This variable flagged children as having potential language delays if, when children were 3 years old, their mothers reported either that they were ‘not yet’ making ‘sentences that are three or four words long’ (an item from the ASQ) or if they reported children as either ‘Not yet talking’, ‘… talking, but you can't understand him/her’, ‘Talking in one-word utterances, such as *milk* or *down*’, or ‘Talking in 2- to 3-word phrases, such as *me got ball* or *give doll*’ (as opposed to either using ‘fairly complete’ or ‘long and complicated’ sentences). This item comes from (and has been validated in) a UK-based twin sample (Dale, Price, Bishop, & Plomin, 2003). Details of internal validation checks of this derived measure are presented in online Supplementary eMethods 3.

To augment the measures addressing motor and language development in specific terms, we included one additional binary variable from the questionnaire administered when children were 3 years old. This was a question asking mothers to report (‘Yes’/”No”) whether ‘others (family, nursery, health visitor) have expressed concerns about your child's development’.

### Assessment for bias and selection effects

We investigated two selection-related biases in our analyses. First, it is possible that there are selection effects associated with inclusion in the early batches of MoBa genotyping, which are enhanced for complete trios. We quantified this by comparing the genotyped sub-sample with the remainder of the full MoBa sample on the phenotypic measures from the main analyses. Second, selective attrition (where drop-out in longitudinal studies is predicted by analysis-relevant variables) also represents a potential source of bias. We quantified this by assessing the extent to which provision of data at each wave was associated with individuals' PGS for ADHD, autism, and schizophrenia.

### Statistical methods

#### Linear regression in multi-group framework

For the continuous variables (age at first walking, age at first words, age at first sentences, rate of language development) we fitted linear regression models in a multi-group framework, with sex as a grouping variable and the various PGS as predictors (one PGS per model). These models were then systematically constrained across sex (with first only the regression coefficients equated across sex, then both the regression coefficients and the intercepts equated across sex, then all model parameters equated). These nested models were compared using χ^2^ difference tests.

#### Probit regression in multi-group framework

For the binary variables (motor delays at 18 months; language delays at 3 years; concern expressed by others by 3 years), we fitted probit regression models in in a multi-group framework, with sex as a grouping variable and the various PGS as predictors (one PGS per model). As with the linear regressions, models were constrained to test for sex differences, and nested models compared using χ^2^ difference tests.

#### Multiple testing

The strength of the evidence for the PGS association in the context of multiple tests being performed was evaluated by using three alpha levels as benchmarks: an uncorrected 0.05 alpha level, an alpha level corrected using the Benjamini−Hochberg method to control the false-discovery rate, and a conservative Bonferroni corrected alpha level of 0.05/42 = 0.001, where 42 corresponds to the maximum possible number of effects of interest from the main analyses (3 PGS×7 outcomes×2 groups).

#### Software and analytic code

All modelling was carried out in R version 3.4.4 using the *lavaan* package (Rosseel, 2012). For the linear regression models, a full information maximum likelihood (FIML) approach was used, meaning that all available data contributed to the model. Maximum likelihood estimation is not available for the probit regression models, meaning that a robust weighted least squares estimator (WLSMV in *lavaan*) with listwise deletion was used. Other R packages used in the project are listed in with citations in online Supplementary eMethods 4.

All analytic code is openly available at https://github.com/psychgen/ndd-prs-milestones-moba. The consent given by the participants does not allow for storage of data on an individual level in repositories or journals. Researchers can apply for access to data for replication purposes via MoBa, in line with their data access policies.

### Descriptive statistics

Descriptive statistics for the main study variables among genotyped individuals are shown, for males and females separately, in Table 1. Males and females differed minimally in terms of their motor development. On average, males spoke their first words [*t* = 6.047 (3234.5), *p* < 0.001] and sentences [*t* = 7.159 (3152.1), *p* < 0.001] later than females, progressing from words to sentences at a slower rate [*t* = 3.995 (3043.6), *p* < 0.001] and were more likely to be reported as having language delays [χ^2^ = 120.13 (1), *p* < 0.001]. Additionally, mothers of boys were also more likely to report that others had expressed concern about their child's development [χ^2^ = 75.966 (1), *p* < 0.001, see online Supplementary eTable 1 for full details of cross-sex comparisons on main study variables). The correlations between PGS were ADHD-autism: 0.21 (95% confidence intervals (CIs) 0.20–0.22); ADHD-SCZ: 0.10 (0.09–0.11); and autism-SCZ: 0.01 (0.00–0.03).

**Table 1.** Descriptive statistics for measures of motor and language development between the ages of 18 months and 5 years in the analytic sub-sample of MoBa

Domain	Variable	Sex	*N*	Mean (months) or proportion	s.d.	min	max	*N*_cases_
Motor	Age of first walking	Male	10 484	12.74	2.05	7	33	
		Female	9475	12.76	2.06	6	35	
	Motor delays at 18 months	Male	10 011	0.03	0.16	0	1	272
		Female	9032	0.03	0.16	0	1	242
Language	Age at first words	Male	1725	13.57	4.58	6	60	
		Female	1563	12.70	3.65	6	36	
	Age at first sentences	Male	1654	20.89	5.99	7	60	
		Female	1523	19.48	5.06	6	48	
	Rate(age_sentences_ – age_words_)	Male	1588	7.44	4.28	1	41	
		Female	1460	6.85	3.83	1	31	
	Language delays at 3 years	Male	7959	0.06	0.23	0	1	444
		Female	7251	0.02	0.14	0	1	153
General	Concern about development	Male	7959	0.05	0.21	0	1	372
		Female	7251	0.02	0.14	0	1	151

*Note* – The difference in data availability for the age at first words variable compared to other measures in Table 1 is due to the version of the 5-year questionnaire containing the measure was only sent to a subset of MoBa participants. To check for relevant systematic differences between those who did *v.* did not receive the age at first words questionnaire at the 5-year wave, we compared the proportion of language delays at 3 years in these two groups, finding minimal differences [0.039 *v.* 0.030, χ^2^ = 4.968 (1), *p* = 0.030].

### Selection and selective attrition

Results from our analyses of selection and selective attrition are presented in online Supplementary eTables 2–4. We found some evidence of selection bias associated with having genotype data available, but limited evidence of ongoing selective attrition associated with neurodevelopmental PGS.

### Main analyses

**Fig. 1.**
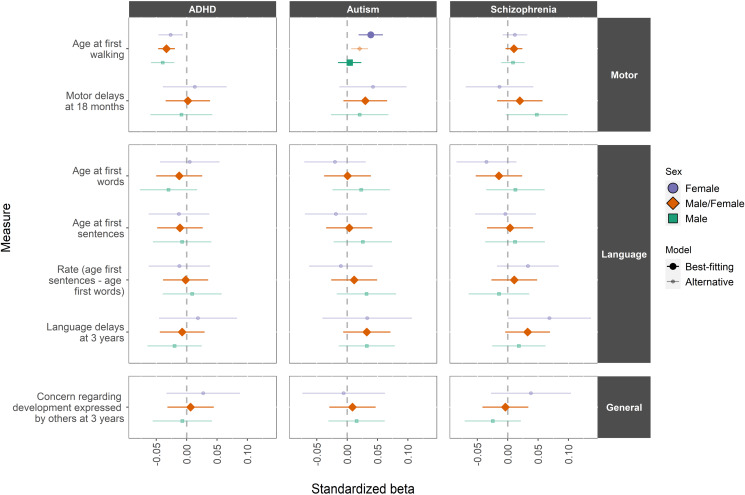
Results from regression models testing effects of PGS for ADHD, Autism, and schizophrenia on the attainment of motor and language developmental milestones. *Note*: estimates from both sex-stratified and constrained models are presented for outcome; the presence of 95% CI bars and darker fill intensity indicate which model provided a better fit to the data; estimates are presented only for the PGS threshold which maximised *R*^2^ in the best-fitting model

The results of the main analyses are presented in Fig. 1. In the figure, standardised beta coefficients from the sex stratified and best-fitting constrained linear/probit models are plotted for each outcome variable regressed on each PGS. The size and opacity of the points indicate which model was preferred in each analysis (sex-stratified or male/females together). The CIs in Fig. 1 correspond to the uncorrected 0.05 alpha level.

In the motor development analyses (Fig. 1; top panel), we found robust evidence that ADHD PGS is associated with younger age at first walking (*β* = −0.033, Bonferroni-adjusted *p* < 0.001) an association that was present in both males and females. Additionally, we observed robust evidence for an association of autism PGS with age at first walking in females only: *β* = 0.039, Bonferroni-adjusted *p* = 0.006). We observed no robust evidence of any association of these PGS on the measure of motor delays at 18 months, nor of schizophrenia PGS being associated with either measure of motor development. Similarly, we found no robust evidence of associations between any neurodevelopmental PGS and outcomes in the language or general development domains. Full model-fitting results for these analyses are presented in online Supplementary eTables 5, 6 and 7, with parameter estimates and raw/adjusted *p* values from best-fitting models in online Supplementary eTable 8.

#### Effect sizes

**Fig. 2.**
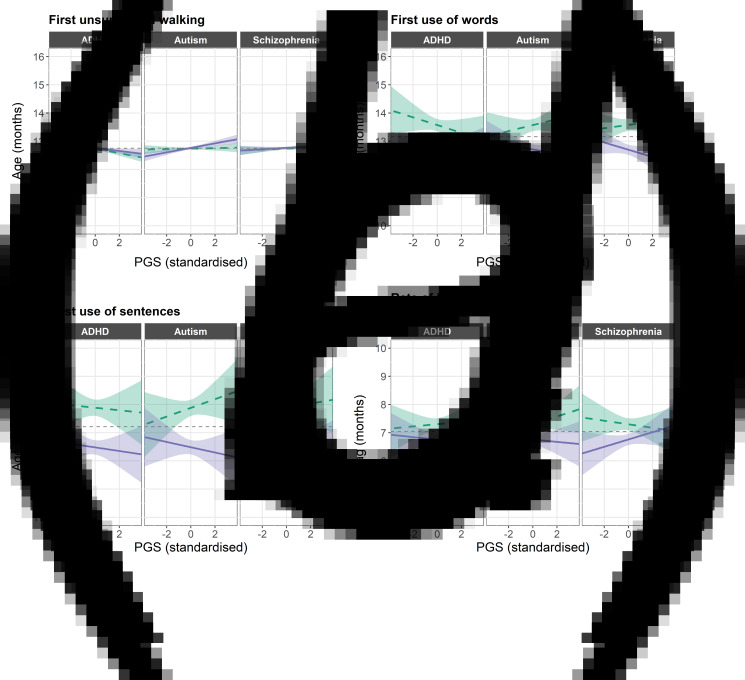
Neurodevelopmental PGS and continuous measures of motor and language development from the MoBa sample. *Note*: *Y*-axes differ but range remains of constant size to facilitate comparison of effect sizes.

Figure 2 shows the relationships between the three neurodevelopmental PGS and, respectively, children's age at first walking, first words, first use of sentences, and rate of language development. The observed associations for ADHD and autism PGS on age at first walking (panel A) were small: a 1SD increase in ADHD polygenic burden was associated with a few days' reduction in the age at which children first walked, while a similar increase in autism PGS translated to later walking by a few days in girls only.

### *Post hoc* sensitivity analyses

To aid interpretation of the results from the main analyses we ran a series of *post hoc* sensitivity analyses. Firstly, we re-ran analyses using a PGS for a non-neurodevelopmental condition (major depressive disorder; Howard et al., 2018)) as a negative control, finding no associations with any of the outcome measures in the study (see online Supplementary eTable 9). Next, we sought to investigate the extent to which the observed associations were driven by individuals who went on to receive a diagnosis of either ADHD or autism. Based on information from the Norwegian Patient Registry, we identified and excluded 1689 individuals in our analytic sample who had received a diagnosis code in the F90 sub-chapter of ICD-10 (‘hyperkinetic disorders’) and 340 individuals F84 sub-chapter (‘pervasive developmental disorders’) as of 2018. This left an analytic sample of *N* = 23 852, and the pattern of results was unchanged in this sample (see online Supplementary eTable 10). Finally, to account for the possibility that an unknown number of individuals from a particular city of Norway within our analytic sample may have had parents who were included in the ADHD GWAS used as the basis of the PGS and that this potential sample overlap could have biased results, we re-ran the analyses with all individuals from this region (Hordaland, including the city of Bergen) excluded. This reduced the analytic sample size to *N* = 22 337 and, again, results from this sensitivity analyses were essentially unchanged from the main analyses (see online Supplementary eTable 11).

## Discussion

We investigated the extent to which timely attainment of early-life developmental milestones was associated with genetic liability to neurodevelopmental disorders. The most robust evidence was observed for an association between genetic liability to ADHD and earlier walking in both boys and girls, and genetic liability to autism and later walking in girls only. We found no robust evidence for associations between genetic risk for neurodevelopmental disorders and language milestone attainment in early life.

ADHD PGS were associated with earlier walking in our sample, in both boys and girls. Our sensitivity analyses showed that these results were not driven exclusively by the presence of individuals who would go on to receive diagnoses of ADHD and related disorders. This suggests that liability for ADHD manifests broadly, in ways that may be neither directly related to ADHD symptomatology nor even necessarily functionally impairing at all, from very early in life. It should be noted that our finding regarding ADHD genetic liability and earlier walking is partly consistent with results from a study in the Danish National Birth Cohort (Lemcke, Parner, Bjerrum, Thomsen, & Lauritsen, 2016), which found an over-representation of early-walkers among ADHD-diagnosed children. However, unlike that study, we did not find evidence of any non-linearity in this relationship (late-walkers were also over-represented in ADHD-diagnosed children in Lemcke et al., 2016) – and further, we show through our sensitivity analyses that this finding is robust among children who do not receive ADHD diagnoses.

We also observed associations between autism PGS and age at first walking. In contrast to those observed for ADHD PGS, these were sex-specific – appearing only in girls – and in the opposite direction, with higher PGS associated with later walking. This reversal of the direction of the effects is noteworthy given the positive (*r* = 0.21) correlation between ADHD and autism PGS. The finding appears to be consistent with the notion that there is a genetic basis to commonly observed associations between delays in motor development and autism (Harris, 2017; West, 2019). However, as with the ADHD PGS results, we confirmed with *post hoc* sensitivity analyses that this pattern was not entirely driven by autism cases within the sample. Moreover, the reason for the sex differentiated nature of the association is unclear. Diagnostic bias in autism may mean that female manifestations are less recognised clinically (Loomes, Hull, & Mandy, 2017). Additionally, motor delays may be a more common early sign in autistic girls compared to autistic boys (Gabis, Attia, Roth-Hanania, & Foss-Feig, 2020). In terms of genetic evidence, rare high-impact *de novo* risk variants for autism are associated both with female sex and later age of walking in autism case samples (Havdahl et al., 2021; Satterstrom et al., 2020). Overall, further studies are needed to establish whether, as is apparent in our results, a similar pattern exists for common inherited genetic risk for autism in the general population – and the extent to which this may be consequential for sex differentiation in clinical manifestations of autism.

We did not find any evidence for associations between neurodevelopmental PGS and measures of the timeliness with which early language milestones were attained. This absence-of-evidence should not be taken as evidence that such effects do not exist, and it should be noted that, for practical reasons detailed further in the limitations section below, none of our analyses using measures of language milestone attainment were as well powered as those using the age-at-first walking outcome. Nonetheless, it is possible that manifestations of genetic liability for neurodevelopmental disorders either emerge later in the domain of language development than for motor development, or are more observable as specific difficulties rather than delayed attainment (as we have shown in this sample elsewhere: see Askeland et al., 2020).

There are some limitations to this work. First, age at first walking and talking were based on maternal recall. Age of walking has been demonstrated to have high reliability when relying on parental recall (Langendonk et al., 2007) and was assessed on two occasions here. However, age of first word/sentence use was assessed only at 5 years, only in a subset of the sample, and may be more imprecise (Majnemer & Rosenblatt, 1994) or subject to bias (Hus, Taylor, & Lord, 2011). It is also possible that discrepancies between reported and actual ages of milestone attainment are not simply due to measurement error, but also recall bias. For example, a child who is more active at the time when the mothers fill in the questionnaire may be recalled as having walked earlier. Second, the sample was subject to some selection associated with study variables. Like all cohort studies, MoBa is more successful at retaining participants with who are healthier and more educated, and this is reflected in the polygenic liabilities of those who responded on the measures of developmental milestones used in these analyses. We cannot rule out some bias in the results as a consequence of this selection bias. Finally, limitations exist around both GWAS and polygenic scoring approaches, and these apply to this work and are no less important for being well-documented in the literature (Tam et al., 2019; Wray et al., 2013). In particular, we note that strong conclusions from direct comparisons across different PGS should be avoided given the differences in the power of the original GWAS on which the PGS are based (as well as in the polygenicity and SNP-heritability of the traits).

## Conclusions

Overall, our findings suggest that common genetic variants associated with neurodevelopmental disorders are also linked to the attainment of early motor developmental milestones. We find no robust evidence for similar relationships with language developmental milestones. The associations did not appear to be driven by clinical cases within the sample, suggesting that polygenic liability for neurodevelopmental disorders manifests – at least in respect of the attainment of early motor developmental milestones – from very early in life in the general population.

## Footnotes

† The notes appear after the main text.

## Footnotes

1 Individuals from MoBa who were identified as having been included in one of the GWAS on which the calculation of the polygenic scores were based (or being children of a parent who had been) were excluded. For one potential source of overlap, only the geographical region of Norway from which MoBa children' parents had potentially been included in the ADHD GWAS was known, so individuals in the analytic sample from this region were removed as a sensitivity analysis.

## Supplementary material

For supplementary material accompanying this paper visit https://doi.org/10.1017/S0033291721003330. click here to view supplementary material
