# Supplementary material for: Developmental milestones in early childhood and genetic liability to neurodevelopmental disorders
Source: Psychol Med. 2021 Sep 21;53(5):1750–8. doi: 10.1017/S0033291721003330 (PMC10106302; doi:10.1017/S0033291721003330)
Supplement: Supplementary file 1 [file S0033291721003330sup001.docx]

Developmental milestones in early childhood and genetic liability to neurodevelopmental disorders - online appendix

[eMethods 1: Supplementary information about genetic data (including quality control) 2](#_Toc76125965)

[Post-imputation data quality control 2](#_Toc76125966)

[Ancestry 2](#_Toc76125967)

[Degree of relatedness 2](#_Toc76125968)

[Principal components 3](#_Toc76125969)

[References 3](#_Toc76125970)

[eMethods 2: Supplementary information about polygenic scoring 3](#_Toc76125971)

[Number of SNPs per threshold for each score: 5](#_Toc76125972)

[Correlations between scores and derived PRS-PCs 6](#_Toc76125973)

[eMethods 3: Internal validation checks of derived measures of motor and language delays 7](#_Toc76125974)

[eMethods 4: R packages used in project 7](#_Toc76125975)

[Package references 8](#_Toc76125976)

[eTable 1. Results of cross-sex comparisons on main study variables 8](#_Toc76125977)

[Selection and selective attrition 9](#_Toc76125978)

[eTable 2. Selection effects in genotyped sample: continuous variables 9](#_Toc76125979)

[eTable 3. Selection effects in genotyped sample: binary variables 9](#_Toc76125980)

[eTable 4. Selective attrition testing results 9](#_Toc76125981)

[eTable 5. Model fitting results for motor development analyses 10](#_Toc76125982)

[eTable 6. Model fitting results for language development analyses 10](#_Toc76125983)

[eTable 7. Model fitting results for concern about development analyses 11](#_Toc76125984)

[eTable 8. Parameter estimates and raw/adjusted p values from best-fitting models 11](#_Toc76125985)

[eTable 9. Sensitivity analysis: MDD PRS.PC as negative control (best-fitting models) 12](#_Toc76125986)

[eTable 10. Sensitvity analysis: removal of autism and ADHD cases 12](#_Toc76125987)

[eTable 11. Sensitivity analysis: removal of participants in region with potential overlap with ADHD GWAS (ADHD PGS results only) 13](#_Toc76125988)

# eMethods 1: Supplementary information about genetic data (including quality control)

This data release is derived from the MoBa Genetics genotype data release 1.0 (n samples=98,110), based on genotype data from four research projects. 33,199 individuals in the NORMENT project were genotyped at deCODE genetics, Reykjavik Iceland (Illumina HumanOmniExpress-24v1.0, Illumina InfiniumOmniExpress-24v1.2, & Illumina Global Screening Array MD v.1.0 + 50k custom OmniExpress overlap content array), 26,990 were genotyped in the ROTTERDAM project at ERASMUS MC, Rotterdam, Netherlands (Illumina Global Screening Array MD v.1.0 array), and 5,410 from the ADHD project (TED) were genotyped at deCODE genetics (Illumina InfiniumOmniExpress-24v1.2), and 32,538 were sampled in the HARVEST sample at Genomics Core Facility, Trondheim, Norway (llumina HumanCoreExome12v1.1 & Illumina HumanCoreExome24v1.0). Pre-imputation QC, phasing and imputation are described [here](https://github.com/folkehelseinstituttet/mobagen/wiki/MoBaGenetics1.0) and in Helgeland et al. (2021).

## Post-imputation data quality control

Individuals with sex-mismatch (derived by comparing genetic sex and reported sex) or individuals with sex-chromosome aneuploidy or those that were not linkable with the phenotypic data were excluded from the analysis (n=508). We checked the dataset for Mendelian errors using PLINK’s –mendel command with thresholds of 1% and 5% for the trio and variant error rate respectively. This excluded 129 individuals and 1293 variants. On average these variants were less precisely imputed, mean INFO=0.88 versus mean INFO=0.97 for the rest of the SNPs. 3,061 individuals were genotyped twice, and 52 individuals were genotyped 3 times. We checked the concordance of SNPs within pairs of duplicated samples, and 2 samples, that were indicated as duplicates by the MoBa data, had very low concordance (pi_hat<0.03), and were excluded. The remaining individuals had high concordance (pi_hat>0.8). Note there were some 2,474 pairs of samples that had concordance between 0.74 and 0.98. These individuals are almost certainly the same individuals and are unlikely to represent sample contamination (this would have been picked up at the genotype calling stage). Therefore this heterogeneity is likely due to the fact that samples for the same individuals are combined from different chips which have not been imputed to the same standard. We excluded 140,767 SNPs which were discordant for more than 5% of duplicated samples. One individual from each pair of duplicates was then dropped at random with a seed.

## Ancestry

We restricted the sample to individuals of “European” ancestry using the first 2 principal components of the MoBa data. The principal components were calculated by merging the MoBa data with the 1000g reference panel and projecting the 1000g PCs onto the MoBa data. We then compared the PC values in the MoBa samples versus each of the populations included in the reference panel. We excluded samples if they had values of PC1 and PC2 that were within the range of the non-European samples in the 1000g reference panel, leading to the exclusion of 668 non-European samples.

## Degree of relatedness

Estimated kinship coefficients using the KING with age to the nearest year included as a covariate. identified 86,175 pairs of known related individuals from the pedigree and a further 10,769 unknown related individuals. Parental relationships were updated on the basis of KING results. Before running KING, we restricted it to an independent set of high frequency SNPs (MAF>0.10, window=3000kb and LD R2 >0.9). KING estimates the relationships of all the individuals in the datasets and reconstructs families. The output is a list of family and within family IDs. KING updated family IDs for 24,022 individuals, and parental relationships for 21,361 individuals. Samples were flagged for exclusion if newly assigned relationships appeared to be errors (375 samples). This included where: parents were less than 15 years older than children, both parents in a family were of the same sex, individuals were identified as MZ twins but linked to different pregnancies, and siblings had different parents or an age gap of more than 25 years. We also flagged for exclusion parent-offspring pairs where the mother or father as identified by KING was different to the genotyped individual specified in the pedigree. This category will include both samples where the sampled partner is not the biological father, and potentially sample mix-ups. Independently within the samples of parents and offspring we used GCTA to select an unrelated subsample using a threshold of IBS < 5% after pruning to an independent set of HAPMAP3 SNPs.

## Principal components

The first 20 principal components were calculated independently for offspring and parents on a subset of the data limited to variants in HAPMAP3, that were pruned for independence using PLINK. We constructed two sets of principal components, the first used the MoBa data and accounts for structure within the data. The second set of principal components was generated using the 1000 genomes reference panel.

A full description of the pipeline for post-imputation quality control applied here will be made available at <https://research-information.bris.ac.uk/en/datasets/> with the title: “MoBa Genetic Data: NIPH PsychGen and MRC IEU post-imputation QC of MoBaGenetics release 1.0”.

## References

Helgeland, Øyvind, Marc Vaudel, Pol Sole-Navais, Christopher Flatley, Julius Juodakis, Jonas Bacelis, Ingvild L. Koløen, Gun Peggy Knudsen, Bente B. Johansson, Per Magnus, Ted Reichborn Kjennerud, Petur B. Juliusson, Camilla Stoltenberg, Oddgeir L. Holmen, Ole A. Andreassen, Bo Jacobsson, Pål R. Njølstad, and Stefan Johansson. 2021. Characterization of the Genetic Architecture of BMI in Infancy and Early Childhood Reveals Age-Specific Effects and Implicates Pathways Involved in Mendelian Obesity. MedRxiv 2021.05.04.21256508. doi: 10.1101/2021.05.04.21256508.

# eMethods 2: Supplementary information about polygenic scoring

A summary of the parameters applied to polygenic scores is provided below (set via the relevant flags in the PRSice software):

-Clumping parameters: window size = 500kb; p-value threshold = 1; r2 threshold= 0.10

-INFO score threshold (for SNPs in original GWAS): 0.9

-Minor allele frequency threshold (for SNPs in original GWAS; available for schizophrenia only): 0.05

-Exclusion of MHC region specified at chr6:25000000-34000000

An abbreviated log file for one of the PRSice runs is provided below to illustrate the implementation of these parameters:

*PRSice 2.3.3 (2020-08-05)*[*https://github.com/choishingwan/PRSice*](https://github.com/choishingwan/PRSice) *(C) 2016-2020 Shing Wan (Sam) Choi and Paul F. O’Reilly
GNU General Public License v3
If you use PRSice in any published work, please cite:
Choi SW, O’Reilly PF.
PRSice-2: Polygenic Risk Score Software for Biobank-Scale Data.
GigaScience 8, no. 7 (July 1, 2019)
2021-06-18 22:25:53
/cluster/p/p471/cluster/common/prsice/PRSice_linux \
    –a1 A1 \
    –a2 A2 \
    –all-score \
    –bar-levels 5e-08,1e-06,1e-05,1e-04,0.001,0.01,0.005,0.1,0.5,1 \
    –base /cluster/p/p471/cluster/common/gwas_sumstats/adhd_eur_jun2017 \
    –base-info* [*INFO:0.9*](INFO:0.9) *\
    –binary-target T \
    –bp BP \
    –chr CHR \
    –clump-kb 500kb \
    –clump-p 1.000000 \
    –clump-r2 0.250000 \
    –fastscore \
    –lower 5e-08 \
    –maf 0.01 \
    –no-default \
    –no-regress \
    –num-auto 22 \
    –or \
    –out /cluster/p/p471/cluster/common/raw_prsice_output/laurie/2021-06-17/maf0.01/clump500_1_0| .25/adhd/adhd \
    –print-snp \
    –pvalue P \
    –seed 1274551647 \
    –snp SNP \
    –stat OR \
    –target /cluster/p/p471/cluster/projects/moba_interim_release_post_imp_qc/qcd_genetic_data_F| eb22/merge.no_batch.noX.geno.mind.hwe \
    –thread 1 \
    –x-range chr6:25000000-34000000

Initializing Genotype file:
/cluster/p/p471/cluster/projects/moba_interim_release_post_imp_qc/qcd_genetic_data_Feb22/merge.no| _batch.noX.geno.mind.hwe
(bed)

Start processing adhd_eur_jun2017
==================================================

Base file:
/cluster/p/p471/cluster/common/gwas_sumstats/adhd_eur_jun2017
Header of file is:
CHR SNP BP A1 A2 INFO OR SE P

8094094 variant(s) observed in base file, with:
64852 variant(s) excluded as they fall within x-range
region(s)
733611 variant(s) with INFO score less than 0.900000
1021160 ambiguous variant(s) excluded
6274471 total variant(s) included from base file

Loading Genotype info from target
==================================================

93211 people (46694 male(s), 46517 female(s)) observed
63415 founder(s) included

2178721 variant(s) not found in previous data
338 variant(s) with mismatch information
4962423 variant(s) included

Calculate MAF and perform filtering on target SNPs
==================================================

2010 variant(s) excluded based on MAF threshold
4960413 variant(s) included

Start performing clumping

Number of variant(s) after clumping : 221840

Start calculating the scores ***

## Number of SNPs per threshold for each score:

| PGS | Threshold | Num_SNP |
| --- | --- | --- |
| adhd | p<5e-08 | 13 |
| adhd | p<1e-06 | 40 |
| adhd | p<1e-05 | 112 |
| adhd | p<1e-04 | 394 |
| adhd | p<0.001 | 1849 |
| adhd | p<0.005 | 5678 |
| adhd | p<0.01 | 9494 |
| adhd | p<0.1 | 51950 |
| adhd | p<0.5 | 161010 |
| adhd | p<1 | 221840 |
| asd | p<5e-08 | 2 |
| asd | p<1e-06 | 14 |
| asd | p<1e-05 | 56 |
| asd | p<1e-04 | 243 |
| asd | p<0.001 | 1338 |
| asd | p<0.005 | 4568 |
| asd | p<0.01 | 7833 |
| asd | p<0.1 | 47682 |
| asd | p<0.5 | 155818 |
| asd | p<1 | 217715 |
| scz | p<5e-08 | 345 |
| scz | p<1e-06 | 671 |
| scz | p<1e-05 | 1285 |
| scz | p<1e-04 | 2700 |
| scz | p<0.001 | 6585 |
| scz | p<0.005 | 13812 |
| scz | p<0.01 | 19457 |
| scz | p<0.1 | 68592 |
| scz | p<0.5 | 168876 |
| scz | p<1 | 220632 |

## Correlations between scores and derived PRS-PCs

ADHD

|  | PRS.PC | p<5e-08 | p<1e-06 | p<1e-05 | p<1e-04 | p<0.001 | p<0.005 | p<0.01 | p<0.1 | p<0.5 | p<1 |
| --- | --- | --- | --- | --- | --- | --- | --- | --- | --- | --- | --- |
| PRS.PC | 1 |  |  |  |  |  |  |  |  |  |  |
| p<5e-08 | 0.47 | 1 |  |  |  |  |  |  |  |  |  |
| p<1e-06 | 0.56 | 0.71 | 1 |  |  |  |  |  |  |  |  |
| p<1e-05 | 0.61 | 0.51 | 0.72 | 1 |  |  |  |  |  |  |  |
| p<1e-04 | 0.68 | 0.37 | 0.49 | 0.68 | 1 |  |  |  |  |  |  |
| p<0.001 | 0.76 | 0.22 | 0.32 | 0.43 | 0.63 | 1 |  |  |  |  |  |
| p<0.005 | 0.82 | 0.16 | 0.24 | 0.32 | 0.48 | 0.74 | 1 |  |  |  |  |
| p<0.01 | 0.82 | 0.14 | 0.21 | 0.29 | 0.43 | 0.66 | 0.89 | 1 |  |  |  |
| p<0.1 | 0.78 | 0.09 | 0.13 | 0.19 | 0.29 | 0.48 | 0.65 | 0.74 | 1 |  |  |
| p<0.5 | 0.75 | 0.07 | 0.11 | 0.16 | 0.24 | 0.41 | 0.58 | 0.66 | 0.91 | 1 |  |
| p<1 | 0.74 | 0.07 | 0.11 | 0.16 | 0.24 | 0.41 | 0.57 | 0.65 | 0.91 | 0.99 | 1 |

Autism

|  | PRS.PC | p<5e-08 | p<1e-06 | p<1e-05 | p<1e-04 | p<0.001 | p<0.005 | p<0.01 | p<0.1 | p<0.5 | p<1 |
| --- | --- | --- | --- | --- | --- | --- | --- | --- | --- | --- | --- |
| PRS.PC | 1 |  |  |  |  |  |  |  |  |  |  |
| p<5e-08 | 0.31 | 1 |  |  |  |  |  |  |  |  |  |
| p<1e-06 | 0.35 | 0.43 | 1 |  |  |  |  |  |  |  |  |
| p<1e-05 | 0.52 | 0.36 | 0.61 | 1 |  |  |  |  |  |  |  |
| p<1e-04 | 0.63 | 0.3 | 0.39 | 0.66 | 1 |  |  |  |  |  |  |
| p<0.001 | 0.76 | 0.21 | 0.24 | 0.43 | 0.63 | 1 |  |  |  |  |  |
| p<0.005 | 0.85 | 0.17 | 0.17 | 0.33 | 0.48 | 0.74 | 1 |  |  |  |  |
| p<0.01 | 0.86 | 0.16 | 0.15 | 0.3 | 0.43 | 0.65 | 0.88 | 1 |  |  |  |
| p<0.1 | 0.85 | 0.11 | 0.1 | 0.2 | 0.3 | 0.46 | 0.64 | 0.73 | 1 |  |  |
| p<0.5 | 0.81 | 0.09 | 0.08 | 0.17 | 0.26 | 0.4 | 0.56 | 0.64 | 0.91 | 1 |  |
| p<1 | 0.81 | 0.09 | 0.08 | 0.17 | 0.26 | 0.39 | 0.56 | 0.64 | 0.9 | 0.99 | 1 |

Schizophrenia

|  | PRS.PC | p<5e-08 | p<1e-06 | p<1e-05 | p<1e-04 | p<0.001 | p<0.005 | p<0.01 | p<0.1 | p<0.5 | p<1 |
| --- | --- | --- | --- | --- | --- | --- | --- | --- | --- | --- | --- |
| PRS.PC | 1 |  |  |  |  |  |  |  |  |  |  |
| p<5e-08 | 0.7 | 1 |  |  |  |  |  |  |  |  |  |
| p<1e-06 | 0.78 | 0.85 | 1 |  |  |  |  |  |  |  |  |
| p<1e-05 | 0.83 | 0.74 | 0.87 | 1 |  |  |  |  |  |  |  |
| p<1e-04 | 0.87 | 0.63 | 0.75 | 0.86 | 1 |  |  |  |  |  |  |
| p<0.001 | 0.9 | 0.53 | 0.63 | 0.73 | 0.85 | 1 |  |  |  |  |  |
| p<0.005 | 0.9 | 0.44 | 0.54 | 0.63 | 0.74 | 0.88 | 1 |  |  |  |  |
| p<0.01 | 0.89 | 0.41 | 0.5 | 0.59 | 0.7 | 0.84 | 0.95 | 1 |  |  |  |
| p<0.1 | 0.82 | 0.32 | 0.39 | 0.47 | 0.56 | 0.69 | 0.81 | 0.86 | 1 |  |  |
| p<0.5 | 0.78 | 0.27 | 0.34 | 0.41 | 0.51 | 0.63 | 0.75 | 0.79 | 0.94 | 1 |  |
| p<1 | 0.77 | 0.27 | 0.34 | 0.41 | 0.5 | 0.63 | 0.74 | 0.79 | 0.94 | 1 | 1 |

# eMethods 3: Internal validation checks of derived measures of motor and language delays

We carried out an internal validation check of the derived measure of motor delays at 18 months in the full MoBa sample, ascertaining that it had a moderately strong relationship (Φ = 0.25, p<0.001) to maternal-reports of children being seen by a specialist for motor problems by age 8, as well as a weaker relationship to similar measures of specialist contact for autism (Φ = 0.08, p<0.001) and ADHD (Φ = 0.08, p<0.001).

We carried out an internal validation check of the derived measure of language delays at 3 years, ascertaining that it had a moderately strong relationship (Φ = 0.31, p<0.001) to maternal-reports of children being seen by a specialist for language problems by age 8, as well as a weaker relationship to similar measures of specialist contact for autism (Φ = 0.08, p<0.001) and ADHD (Φ = 0.11, p<0.001).

# eMethods 4: R packages used in project

#

- base (R Core Team 2018b)
- foreign (R Core Team 2018a)
- grateful (Rodriguez-Sanchez 2018)
- gtools (Warnes, Bolker, and Lumley 2018)
- lavaan (Rosseel 2012)
- MASS (Venables and Ripley 2002)
- patchwork (Pedersen 2020)
- powerAnalysis (Fan 2017)
- psych (Revelle 2018)
- RColorBrewer (Neuwirth 2014)
- semPlot (Epskamp 2019)
- semTools (Jorgensen et al. 2020)
- tidyverse (Wickham 2017)
- yarrr (Phillips 2017)

## Package references

Epskamp, Sacha. 2019. *SemPlot: Path Diagrams and Visual Analysis of Various Sem Packages’ Output*. <https://CRAN.R-project.org/package=semPlot>.

Fan, Felix Yanhui. 2017. *PowerAnalysis: Power Analysis in Experimental Design*. <https://CRAN.R-project.org/package=powerAnalysis>.

Jorgensen, Terrence D., Sunthud Pornprasertmanit, Alexander M. Schoemann, and Yves Rosseel. 2020. *semTools: Useful Tools for Structural Equation Modeling*. <https://CRAN.R-project.org/package=semTools>.

Neuwirth, Erich. 2014. *RColorBrewer: ColorBrewer Palettes*. <https://CRAN.R-project.org/package=RColorBrewer>.

Pedersen, Thomas Lin. 2020. *Patchwork: The Composer of Plots*. <https://CRAN.R-project.org/package=patchwork>.

Phillips, Nathaniel. 2017. *Yarrr: A Companion to the E-Book “Yarrr!: The Pirate’s Guide to R”*. <https://CRAN.R-project.org/package=yarrr>.

R Core Team. 2018a. *Foreign: Read Data Stored by ’Minitab’, ’S’, ’Sas’, ’Spss’, ’Stata’, ’Systat’, ’Weka’, ’dBase’, ...* <https://CRAN.R-project.org/package=foreign>.

———. 2018b. *R: A Language and Environment for Statistical Computing*. Vienna, Austria: R Foundation for Statistical Computing. <https://www.R-project.org/>.

Revelle, William. 2018. *Psych: Procedures for Psychological, Psychometric, and Personality Research*. Evanston, Illinois: Northwestern University. <https://CRAN.R-project.org/package=psych>.

Rodriguez-Sanchez, Francisco. 2018. *Grateful: Facilitate Citation of R Packages*. <https://github.com/Pakillo/grateful>.

Rosseel, Yves. 2012. “lavaan: An R Package for Structural Equation Modeling.” *Journal of Statistical Software* 48 (2): 1–36. <http://www.jstatsoft.org/v48/i02/>.

Venables, W. N., and B. D. Ripley. 2002. *Modern Applied Statistics with S*. Fourth. New York: Springer. <http://www.stats.ox.ac.uk/pub/MASS4>.

Warnes, Gregory R., Ben Bolker, and Thomas Lumley. 2018. *Gtools: Various R Programming Tools*. <https://CRAN.R-project.org/package=gtools>.

Wickham, Hadley. 2017. *Tidyverse: Easily Install and Load the ’Tidyverse’*. <https://CRAN.R-project.org/package=tidyverse>.

# eTable 1. Results of cross-sex comparisons on main study variables

| Measure | Female | Male | Difference | Test statistic | p |
| --- | --- | --- | --- | --- | --- |
| Age at first walking | 12.764 | 12.737 | 0.026 | 0.910 | 0.3628 |
| Age at first words | 12.704 | 13.574 | -0.869 | -6.047 | <0.001 |
| Age at first sentences | 19.481 | 20.887 | -1.406 | -7.159 | <0.001 |
| Rate | 6.852 | 7.439 | -0.587 | -3.995 | <0.001 |
| Motor delays at 18 months | 0.027 | 0.027 | 0.000 | 0.013 | 0.9082 |
| Language delays at 3 years | 0.021 | 0.056 | -0.035 | 120.132 | <0.001 |
| Concern about development | 0.021 | 0.047 | -0.026 | 75.966 | <0.001 |

# Selection and selective attrition

To ascertain the representativeness of our genotyped sub-sample with respect to the MoBa sample as a whole, we compared the sub-sample to the remainder of MoBa (Nfirst walking = 59964; Nmotor delays = 57258; Nfirst words = 8094; Nlanguage delays = 43542; Nconcern = 43542) in terms of the main analytic variables. No selection effects were found for the continuous variables (age at first walking and age at first words). For the binary variables (motor/language delays, concern about development), we did observe some evidence for small selection effects associated with having genotype data available. For example, among both males and females the proportion of individuals with language delays was higher in those without genotype data available. The selection testing results are presented in full in eTables 2-3.

# eTable 2. Selection effects in genotyped sample: continuous variables

| Variable | sex | mean_Geno | mean_noGeno | diff | lower.ci | upper.ci | pval |
| --- | --- | --- | --- | --- | --- | --- | --- |
| age_1st_wlk | Female | 12.764 | 12.821 | 0.057 | -0.105 | -0.009 | 0.020 |
| age_1st_wlk | Male | 12.737 | 12.818 | 0.080 | -0.126 | -0.034 | <0.001 |
| age_1st_wds | Female | 12.704 | 12.877 | 0.172 | -0.391 | 0.047 | 0.123 |
| age_1st_wds | Male | 13.574 | 13.633 | 0.059 | -0.317 | 0.199 | 0.652 |
| age_1st_sns | Female | 19.481 | 19.663 | 0.182 | -0.488 | 0.124 | 0.244 |
| age_1st_sns | Male | 20.887 | 21.055 | 0.168 | -0.513 | 0.176 | 0.339 |

# eTable 3. Selection effects in genotyped sample: binary variables

| Variable | sex | prop_Geno | prop_noGeno | diff_prop | lower.ci | upper.ci | pval |
| --- | --- | --- | --- | --- | --- | --- | --- |
| mot | Male | 0.027 | 0.032 | -0.004 | -0.008 | -0.001 | 0.031 |
| mot | Female | 0.027 | 0.032 | -0.005 | -0.009 | -0.001 | 0.020 |
| lang | Male | 0.056 | 0.061 | -0.006 | -0.012 | 0.000 | 0.078 |
| lang | Female | 0.021 | 0.025 | -0.004 | -0.008 | 0.000 | 0.070 |
| conc | Male | 0.047 | 0.048 | -0.002 | -0.007 | 0.004 | 0.552 |
| conc | Female | 0.021 | 0.027 | -0.006 | -0.011 | -0.002 | 0.003 |

We also tested for evidence of selective attrition by comparing the average polygenic burden (using the PGS-PCs for each of ADHD, autism, and schizophrenia that were derived for the analyses) among those providing versus not providing data at each wave of measurement. In general, PGS for all traits were slightly lower in those with data available than those without, but this pattern was not universal, and there was no evidence that differences increased with time. Results from the selective attrition testing are available in eTable 4.

# eTable 4. Selective attrition testing results

| PGS | wave | mean_data | mean_nodata | diff | lower.ci | upper.ci | pval |
| --- | --- | --- | --- | --- | --- | --- | --- |
| adhd | 18m | -0.016 | 0.059 | 0.075 | -0.107 | -0.042 | <0.001 |
| adhd | 3yr | -0.024 | 0.046 | 0.070 | -0.099 | -0.041 | <0.001 |
| adhd | 5yr | -0.022 | 0.036 | 0.058 | -0.086 | -0.029 | <0.001 |
| asd | 18m | 0.008 | -0.010 | -0.019 | -0.013 | 0.051 | 0.251 |
| asd | 3yr | 0.016 | -0.016 | -0.032 | 0.003 | 0.061 | 0.028 |
| asd | 5yr | 0.014 | -0.010 | -0.025 | -0.004 | 0.053 | 0.089 |
| scz | 18m | -0.022 | 0.028 | 0.049 | -0.082 | -0.017 | 0.003 |
| scz | 3yr | -0.033 | 0.028 | 0.061 | -0.090 | -0.032 | <0.001 |
| scz | 5yr | -0.039 | 0.030 | 0.069 | -0.098 | -0.041 | <0.001 |

# eTable 5. Model fitting results for motor development analyses

| Variable | PGS | model | AIC | Chisq diff | Df diff | pLRT |
| --- | --- | --- | --- | --- | --- | --- |
| age_1st_wlk | ADHD | Sex diffs | 85350.29 |  |  |  |
| age_1st_wlk | ADHD | Constrained | 85349.14 | 0.853 | 1 | 0.356 |
| age_1st_wlk | ASD | Sex diffs | 85358.65 |  |  |  |
| age_1st_wlk | ASD | Constrained | 85362.69 | 6.035 | 1 | 0.014 |
| age_1st_wlk | SCZ | Sex diffs | 85371.04 |  |  |  |
| age_1st_wlk | SCZ | Constrained | 85369.09 | 0.052 | 1 | 0.819 |
| bin_mot_18m | ADHD | Sex diffs |  |  |  |  |
| bin_mot_18m | ADHD | Constrained |  | 0.349 | 1 | 0.550 |
| bin_mot_18m | ASD | Sex diffs |  |  |  |  |
| bin_mot_18m | ASD | Constrained |  | 0.362 | 1 | 0.550 |
| bin_mot_18m | SCZ | Sex diffs |  |  |  |  |
| bin_mot_18m | SCZ | Constrained |  | 2.512 | 1 | 0.110 |

Notes: AIC (Akaike’s Information Criterion) not available for probit regression models; pLRT = raw p value from likelihood ratio test of sex constrained versus sex difference models;

# eTable 6. Model fitting results for language development analyses

| Variable | PGS | model | AIC | Chisq diff | Df diff | pLRT |
| --- | --- | --- | --- | --- | --- | --- |
| age_1st_wds | ADHD | Sex diffs | 18635.06 |  |  |  |
| age_1st_wds | ADHD | Constrained | 18634.24 | 1.178 | 1 | 0.278 |
| age_1st_wds | ASD | Sex diffs | 18635.13 |  |  |  |
| age_1st_wds | ASD | Constrained | 18634.65 | 1.517 | 1 | 0.218 |
| age_1st_wds | SCZ | Sex diffs | 18634.46 |  |  |  |
| age_1st_wds | SCZ | Constrained | 18634.10 | 1.638 | 1 | 0.201 |
| age_1st_sns | ADHD | Sex diffs | 19889.85 |  |  |  |
| age_1st_sns | ADHD | Constrained | 19887.86 | 0.010 | 1 | 0.921 |
| age_1st_sns | ASD | Sex diffs | 19888.59 |  |  |  |
| age_1st_sns | ASD | Constrained | 19888.16 | 1.571 | 1 | 0.210 |
| age_1st_sns | SCZ | Sex diffs | 19889.94 |  |  |  |
| age_1st_sns | SCZ | Constrained | 19888.15 | 0.214 | 1 | 0.644 |
| diff_1st_wds_sns | ADHD | Sex diffs | 17587.96 |  |  |  |
| diff_1st_wds_sns | ADHD | Constrained | 17586.29 | 0.337 | 1 | 0.561 |
| diff_1st_wds_sns | ASD | Sex diffs | 17586.53 |  |  |  |
| diff_1st_wds_sns | ASD | Constrained | 17585.95 | 1.420 | 1 | 0.233 |
| diff_1st_wds_sns | SCZ | Sex diffs | 17586.34 |  |  |  |
| diff_1st_wds_sns | SCZ | Constrained | 17585.98 | 1.645 | 1 | 0.200 |
| bin_lang_3yr | ADHD | Sex diffs |  |  |  |  |
| bin_lang_3yr | ADHD | Constrained |  | 0.945 | 1 | 0.330 |
| bin_lang_3yr | ASD | Sex diffs |  |  |  |  |
| bin_lang_3yr | ASD | Constrained |  | 0.103 | 1 | 0.750 |
| bin_lang_3yr | SCZ | Sex diffs |  |  |  |  |
| bin_lang_3yr | SCZ | Constrained |  | 1.431 | 1 | 0.230 |

Notes: AIC (Akaike’s Information Criterion) not available for probit regression models; pLRT = raw p value from likelihood ratio test of sex constrained versus sex difference models;

# eTable 7. Model fitting results for concern about development analyses

| Variable | PGS | model | Chisq diff | Df diff | pLRT |
| --- | --- | --- | --- | --- | --- |
| Concern | ADHD | Sex diffs |  |  |  |
| Concern | ADHD | Constrained | 0.760 | 1 | 0.380 |
| Concern | ASD | Sex diffs |  |  |  |
| Concern | ASD | Constrained | 0.312 | 1 | 0.580 |
| Concern | SCZ | Sex diffs |  |  |  |
| Concern | SCZ | Constrained | 2.249 | 1 | 0.130 |

Notes: pLRT = raw p value from likelihood ratio test of sex constrained versus sex difference models;

# eTable 8. Parameter estimates and raw/adjusted p values from best-fitting models

| PGS | var | Sex | std.beta | raw_pval | fdr_pval | bonf_pval |
| --- | --- | --- | --- | --- | --- | --- |
| ADHD | Age at first walking | Male/Female | -0.033 | 0.000 | 0.000 | 0.000 |
| ASD | Age at first walking | Male | 0.004 | 0.671 | 0.974 | 1.000 |
| ASD | Age at first walking | Female | 0.039 | 0.000 | 0.002 | 0.008 |
| Schizophrenia | Age at first walking | Male/Female | 0.010 | 0.147 | 0.611 | 1.000 |
| ADHD | Age at first words | Male/Female | -0.012 | 0.525 | 0.974 | 1.000 |
| ASD | Age at first words | Male/Female | 0.001 | 0.974 | 0.974 | 1.000 |
| Schizophrenia | Age at first words | Male/Female | -0.014 | 0.458 | 0.974 | 1.000 |
| ADHD | Age at first sentences | Male/Female | -0.011 | 0.564 | 0.974 | 1.000 |
| ASD | Age at first sentences | Male/Female | 0.003 | 0.858 | 0.974 | 1.000 |
| Schizophrenia | Age at first sentences | Male/Female | 0.004 | 0.839 | 0.974 | 1.000 |
| ADHD | Rate (age first sentences - age first words) | Male/Female | -0.002 | 0.929 | 0.974 | 1.000 |
| ASD | Rate (age first sentences - age first words) | Male/Female | 0.012 | 0.551 | 0.974 | 1.000 |
| Schizophrenia | Rate (age first sentences - age first words) | Male/Female | 0.011 | 0.573 | 0.974 | 1.000 |
| ADHD | Motor delays at 18 months | Male/Female | 0.002 | 0.913 | 0.974 | 1.000 |
| ASD | Motor delays at 18 months | Male/Female | 0.030 | 0.105 | 0.505 | 1.000 |
| Schizophrenia | Motor delays at 18 months | Male/Female | 0.020 | 0.296 | 0.938 | 1.000 |
| ADHD | Language delays at 3 years | Male/Female | -0.007 | 0.696 | 0.974 | 1.000 |
| ASD | Language delays at 3 years | Male/Female | 0.032 | 0.103 | 0.505 | 1.000 |
| Schizophrenia | Language delays at 3 years | Male/Female | 0.033 | 0.081 | 0.505 | 1.000 |
| ADHD | Concern regarding development expressed by others at 3 years | Male/Female | 0.006 | 0.739 | 0.974 | 1.000 |
| ASD | Concern regarding development expressed by others at 3 years | Male/Female | 0.009 | 0.654 | 0.974 | 1.000 |
| Schizophrenia | Concern regarding development expressed by others at 3 years | Male/Female | -0.004 | 0.844 | 0.974 | 1.000 |

# eTable 9. Sensitivity analysis: MDD PRS.PC as negative control (best-fitting models)

| PGS | var | Sex | std.beta | raw_pval | fdr_pval | bonf_pval |
| --- | --- | --- | --- | --- | --- | --- |
| MDD | Age at first walking | Male/Female | 0.000 | 0.946 | 0.974 | 1 |
| MDD | Age at first words | Male/Female | -0.006 | 0.746 | 0.974 | 1 |
| MDD | Age at first sentences | Male/Female | 0.006 | 0.773 | 0.974 | 1 |
| MDD | Rate (age first sentences - age first words) | Male/Female | 0.018 | 0.356 | 0.938 | 1 |
| MDD | Motor delays at 18 months | Male/Female | 0.018 | 0.353 | 0.938 | 1 |
| MDD | Language delays at 3 years | Male/Female | -0.033 | 0.067 | 0.505 | 1 |
| MDD | Concern regarding development expressed by others at 3 years | Male/Female | -0.023 | 0.244 | 0.885 | 1 |

# eTable 10. Sensitvity analysis: removal of autism and ADHD cases

| PGS | var | Sex | std.beta | raw_pval | fdr_pval | bonf_pval |
| --- | --- | --- | --- | --- | --- | --- |
| ADHD | Age at first walking | Male/Female | -0.032 | 0.000 | 0.001 | 0.001 |
| ASD | Age at first walking | Male | -0.001 | 0.928 | 0.961 | 1.000 |
| ASD | Age at first walking | Female | 0.040 | 0.000 | 0.004 | 0.016 |
| Schizophrenia | Age at first walking | Male/Female | 0.015 | 0.046 | 0.412 | 1.000 |
| ADHD | Age at first words | Male/Female | -0.001 | 0.976 | 0.976 | 1.000 |
| ASD | Age at first words | Male/Female | 0.009 | 0.665 | 0.961 | 1.000 |
| Schizophrenia | Age at first words | Male/Female | -0.007 | 0.738 | 0.961 | 1.000 |
| ADHD | Age at first sentences | Male/Female | -0.004 | 0.837 | 0.961 | 1.000 |
| ASD | Age at first sentences | Male/Female | 0.016 | 0.447 | 0.961 | 1.000 |
| Schizophrenia | Age at first sentences | Male/Female | 0.008 | 0.696 | 0.961 | 1.000 |
| ADHD | Rate (age first sentences - age first words) | Male/Female | -0.004 | 0.859 | 0.961 | 1.000 |
| ASD | Rate (age first sentences - age first words) | Male/Female | 0.016 | 0.456 | 0.961 | 1.000 |
| Schizophrenia | Rate (age first sentences - age first words) | Male/Female | 0.010 | 0.645 | 0.961 | 1.000 |
| ADHD | Motor delays at 18 months | Male/Female | 0.011 | 0.570 | 0.961 | 1.000 |
| ASD | Motor delays at 18 months | Male/Female | 0.029 | 0.144 | 0.597 | 1.000 |
| Schizophrenia | Motor delays at 18 months | Male/Female | 0.021 | 0.305 | 0.957 | 1.000 |
| ADHD | Language delays at 3 years | Male/Female | -0.009 | 0.659 | 0.961 | 1.000 |
| ASD | Language delays at 3 years | Male/Female | 0.038 | 0.071 | 0.412 | 1.000 |
| Schizophrenia | Language delays at 3 years | Male/Female | 0.030 | 0.139 | 0.597 | 1.000 |
| ADHD | Concern regarding development expressed by others at 3 years | Male/Female | 0.003 | 0.877 | 0.961 | 1.000 |
| ASD | Concern regarding development expressed by others at 3 years | Male/Female | 0.008 | 0.707 | 0.961 | 1.000 |
| Schizophrenia | Concern regarding development expressed by others at 3 years | Male/Female | -0.002 | 0.928 | 0.961 | 1.000 |

# eTable 11. Sensitivity analysis: removal of participants in region with potential overlap with ADHD GWAS (ADHD PGS results only)

| PGS | var | Sex | std.beta | raw_pval | fdr_pval | bonf_pval |
| --- | --- | --- | --- | --- | --- | --- |
| ADHD | Age at first walking | Male/Female | -0.036 | 0.000 | 0.000 | 0 |
| ADHD | Age at first words | Male/Female | -0.023 | 0.250 | 0.858 | 1 |
| ADHD | Age at first sentences | Male/Female | -0.012 | 0.529 | 0.887 | 1 |
| ADHD | Rate (age first sentences - age first words) | Male/Female | 0.007 | 0.705 | 0.887 | 1 |
| ADHD | Motor delays at 18 months | Male/Female | 0.009 | 0.651 | 0.887 | 1 |
| ADHD | Language delays at 3 years | Male/Female | 0.017 | 0.427 | 0.887 | 1 |
| ADHD | Concern regarding development expressed by others at 3 years | Male/Female | 0.023 | 0.313 | 0.858 | 1 |
